# A combination of silica and cigarette smoke extract exacerbates lung fibrosis: Unveiling a harmful synergy

**DOI:** 10.1371/journal.pone.0330762

**Published:** 2025-08-22

**Authors:** Fahad Alsohime, Narjes Saheb Sharif-Askari, Nasser Saleh Alharbi, Mohammed Faraj Ayad Alosaimi, Rabih Halwani

**Affiliations:** 1 Pediatric Department, College of Medicine, King Saud University, Riyadh, Saudi Arabia; 2 Pediatric critical care unit, King Saud University Medical City, Riyadh, Saudi Arabia; 3 Research Institute for Medical and Health Sciences, University of Sharjah, Sharjah, United Arab Emirates; 4 Department of Clinical Sciences, College of Medicine, University of Sharjah, Sharjah, United Arab Emirates; 5 Immunology Research Laboratory, College of Medicine, King Saud University, Riyadh, Saudi Arabia,; 6 Health and well being sector, NEOM, Tabuk, Saudi Arabia; University of Missouri-Columbia, UNITED STATES OF AMERICA

## Abstract

Smoking could potentiate the profibrotic effects of silica in the lungs, including increasing the risk of cancer and silicosis. Crystalline silica-induced silicosis has been associated with lung fibrosis. Moreover, smoking is strongly linked with an increased risk of idiopathic pulmonary fibrosis. Although pulmonary fibrosis is a recognized feature in asthma airway modeling, the effects of cigarette smoke and silica, both individually and together, have not been studied. We examined the effect of cigarette smoke extract (CSE) on silica-induced fibrosis in asthmatic patients and healthy individuals by using fibroblasts from both groups. Cigarette smoke enhanced the fibrotic effects of silica in healthy and asthmatic lung fibroblasts. Healthy fibroblasts exhibited low baseline levels of fibrotic proteins. However, exposure to CSE and silica significantly increased extracellular matrix (ECM) markers. Asthmatic fibroblasts, with higher baseline levels of these markers, showed even greater upregulation upon exposure. The combination of silica and cigarette smoke also promoted collagen deposition and upregulated levels of matrix metalloproteinases (MMPs) and their inhibitors (TIMP-1 and TIMP-2) in asthmatic fibroblasts. Cessation of smoking and control of silica exposure are essential for reducing lung inflammation and fibrosis. Additionally, therapeutic targets should be investigated for their protective effects against these toxins.

## Introduction

Crystalline silica is a ubiquitous mineral found in the Earth’s crust and is a primary constituent of sand and soil. Exposure to crystalline silica can occur from various sources, including natural phenomena such as volcanic eruptions, dust storms, and sandstorms, as well as from industries like metal mining and construction. Sands and dust mainly consist of crystalline silica, which constitutes between 49.2% and 67.1% of their composition [1,2]. The U.S. Occupational Safety and Health Administration [3] recognizes crystalline silica exposure as an occupational hazard. Prolonged exposure to silica dust results in an increased risk of mortality among patients with various pulmonary diseases, such as silicosis [4], chronic obstructive lung disease (COLD) [5], and lung cancer [6]. Notably, desert dust storms have been linked to an increased risk of asthma exacerbation and hospitalization [7–11]. Communities affected by desert dust storms, such as those in the Middle East and the Caribbean, show a higher incidence of asthma [12]. This highlights the importance of implementing public health measures during dust storms to alleviate the burden on healthcare services.

Cigarette smoking is a significant public health issue that has been known to impact the severity of asthma. Understanding the interplay between smoking and asthma pathogenesis is crucial, as it may modify the response to environmental triggers, including allergens and crystalline silica. Globally, around half of the adult asthma population are current or former smokers [13], of whom 18% have severe asthma [14]. In addition to primary smoking exposure, secondhand smoke exposure can trigger asthma and exacerbate symptoms [15–18]. This subgroup experiences increased exacerbations compared to those with mild or moderate asthma [14,19], alongside a higher incidence of airway and parenchymal abnormalities in smoking asthmatics compared to nonsmokers [19].

Moreover, human studies underscore an association between smoking exposure and increased mortality risk in individuals exposed to silica dust, primarily due to the exacerbation of silica-induced lung toxicity [20–23]. A recent study has emphasized the deleterious effects of combined cigarette smoke and silica exposure, demonstrating exacerbated pulmonary toxicity in rats compared to exposure to each agent alone [24].

It is noteworthy that the individual effects of cigarette smoke and silica on fibrosis have been studied. Crystalline silica-induced silicosis has been associated with lung fibrosis [25]. Moreover, smoking is strongly linked with an increased risk of Idiopathic Pulmonary Fibrosis [26]. While pulmonary fibrosis is a prominent feature of asthma [27], the individual and combined impacts of cigarette smoke and silica on asthma have not been explored. This is the first study aimed to investigate the combined effects of cigarette smoke and silica on fibrosis in asthmatic fibroblasts from asthmatic and healthy individuals. These findings may help guide future clinical care and occupational health practices to reduce preventable lung damage in high-risk groups.

## Methods

### Fibroblast cell culture

Primary lung fibroblasts, from three healthy controls and three severe asthmatic patients, were obtained from Lonza (Switzerland). They were cultured in DMEM/F12 with 10% FBS and 1% penicillin/streptomycin, maintained in a humidified incubator at 37 °C with 5% CO2. Experiments were performed at matched passages, with a maximum of eight repetitions.

### Ethics statement

The healthy and asthmatic human fibroblast cells used in this study were purchased from Lonza. As the cells were obtained from a commercial source and not directly from human participants, this study did not require ethics committee approval.

### Cigarette smoke extract preparation

Marlboro Red cigarettes (1.1 mg nicotine, 15 mg tar, 15 mg carbon monoxide) were used to prepare cigarette smoke extract (CSE) via a pump-assisted bubbling method as previously described [28]. Briefly, one cigarette was bubbled through 20 mL of PBS to create 100% CSE, which was then diluted for cell culture experiments. Fibroblasts were stimulated using the same CSE batch to reduce variability.

### Fibroblast treatment

Once fibroblasts reached 70% confluence, they were exposed to 10% CSE for 15 minutes and then stimulated for 24 hours with 50 μg/mL of silica in fresh medium. The silica particles, comprising 99% silica and ranging from 1 to 5 μm, were heated for 2 hours at 200 °C to inactivate any endotoxins (S5631, Sigma-Aldrich, St. Louis, MO).

### CellTiter-Glo luminescent cell viability assay

Fibroblasts were stimulated for 24 h to determine their viability using the CellTiter-Glo kit, following the manufacturer’s instructions (Promega). Triplicate assays were conducted, with data representing three independent experiments from three unique donors in each group.

### Quantitative real-timepolymerase chain reaction (qRT-PCR)

qRT-PCR was performed using RNA extracted from fibroblasts via the Trizol method as previously described [28]. cDNA synthesis was carried out with the High-Capacity cDNA Reverse Transcription Kit, followed by amplification with Hot FirePol EvaGreen qRT-PCR SuperMix on a QuantStudio 5 system. Gene expression was analyzed using the ΔΔCT method, normalized to 18s rRNA, and expressed as fold change relative to unstimulated controls. The primers are listed in Table 1.

**Table 1:** List of primer sequences used in qRT-PCR.

Genes	Forward primer sequence (5′-3′)	Reverse primer sequence (5′-3′)
*TGF-β*	AAATTGAGGGCTTTCGCCTTA	GAACCCGTTGATGTCCACTTG
*α-SMA*	CTTCGTGTTGCCCCTGAAGAG	GCATAGAGAGACAGCACCGC
*COL1A1*	GATTGACCCCAACCAAGGCTG	GCCGAACCAGACATGCCTC
*COL3A1*	GATCAGGCCAGTGGAAATG	GTGTGTTTCGTGCAACCATC
*18s*	CTACCACATCCAAGGAAGCA	TTTTTCGTCACTACCTCCCCG

### Western blot

Western blotting was performed on fibroblast protein lysates extracted with RIPA buffer, supplemented with 1 mM phenylmethylsulfonyl fluoride (Sigma-Aldrich, Germany) and 1x Protease Inhibitor Cocktail (Sigma-Aldrich, Germany). Protein concentrations were measured using ThermoScientific Pierce BCA Protein Assay Kit (ThermoFisher Scientific, US). Proteins were separated on an 8.5% SDS-PAGE gel, transferred to nitrocellulose membranes, and incubated with primary antibodies: anti-Fibronectin/FN1 (#26836, 1:1000), anti-COL1A1 (#66948, 1:1000), anti-αSmooth Muscle Actin/αSMA (#19245, 1:1000), anti-MMP2 (#40994, 1:1000), anti-MMP1 (#54376, 1:1000), anti-TIMP2 (#5738, 1:1000) and anti-GAPDH (#2118, 1:1000) (Cell Signaling Technologies, Danvers, MA), anti-COL3A1 (ab7778, 1:1000) (Abcam, Sydney, Australia), anti-COL5A1 (SAB4500384, 1:1000) (Sigma-Aldrich, Germany), anti-TIMP1 (MA1–773, 1:1000) and anti-MMP9 (MA5–15886, 1:1000) (Invitrogen). Horseradish peroxidase-conjugated secondary antibodies were used for detection. Protein bands were visualized using the Sapphire™ NIR-Q Imager (Azure Biosystems, Dublin, US) and quantified using ImageJ software.

### Statistical analysis

Data are presented as mean ± standard error of the mean (SEM) and analyzed using GraphPad Prism 8.4 (GraphPad Software, Inc., La Jolla, CA, USA). Statistical significance was determined by one-way ANOVA with a Bonferroni post hoc test for multiple comparisons, with a p-value < 0.05 considered significant.

## Results

### Cigarette smoke did not alter the cell viability in silica-exposed lung fibroblasts

**Fig 1:**
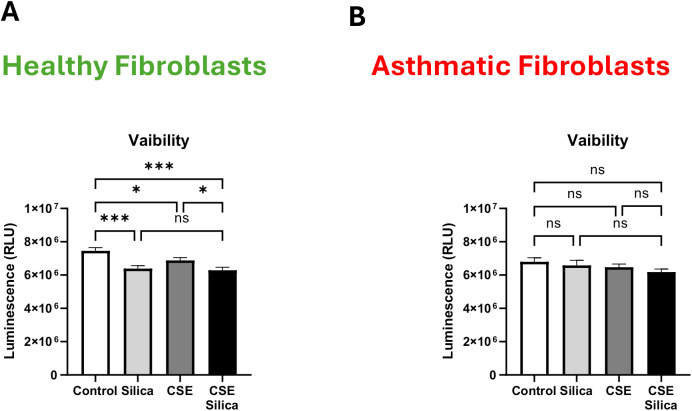
Cigarette smoke did not alter the cell viability in silica-exposed lung fibroblasts. CellTiter-Glo ® luminescence assay was performed to detect the cell viability in healthy **(A)** and asthmatic **(B)** lung fibroblasts exposed to 50 μg/mL Silica microparticles and 10% cigarette smoke extract (CSE). Data representative of 3 independent experiments from 2 unique donors in each group. Results are presented as mean (± SEM). The values were compared across the different groups using one-way ANOVA with a Bonferroni post hoc test for multiple comparisons. ns, non-significant. **p < 0.01.

We first examined the effect of silica exposure on lung fibroblasts and found that it significantly reduced the viability of healthy fibroblasts (p < 0.001) but had no effect on asthmatic fibroblasts. We then assessed the effect of CSE on the viability of silica-exposed fibroblasts and observed that cigarette smoke did not further reduce cell viability in either group (Fig 1A–1B). These results indicate that silica impairs the viability of healthy, but not asthmatic, fibroblasts, and that co-exposure with cigarette smoke does not exacerbate this effect in either cell type. Data are representative of three independent experiments using fibroblasts from two unique donors per group.

### Cigarette smoke enhances the expression of fibrotic markers in silica-stimulated lung fibroblasts

**Fig 2:**
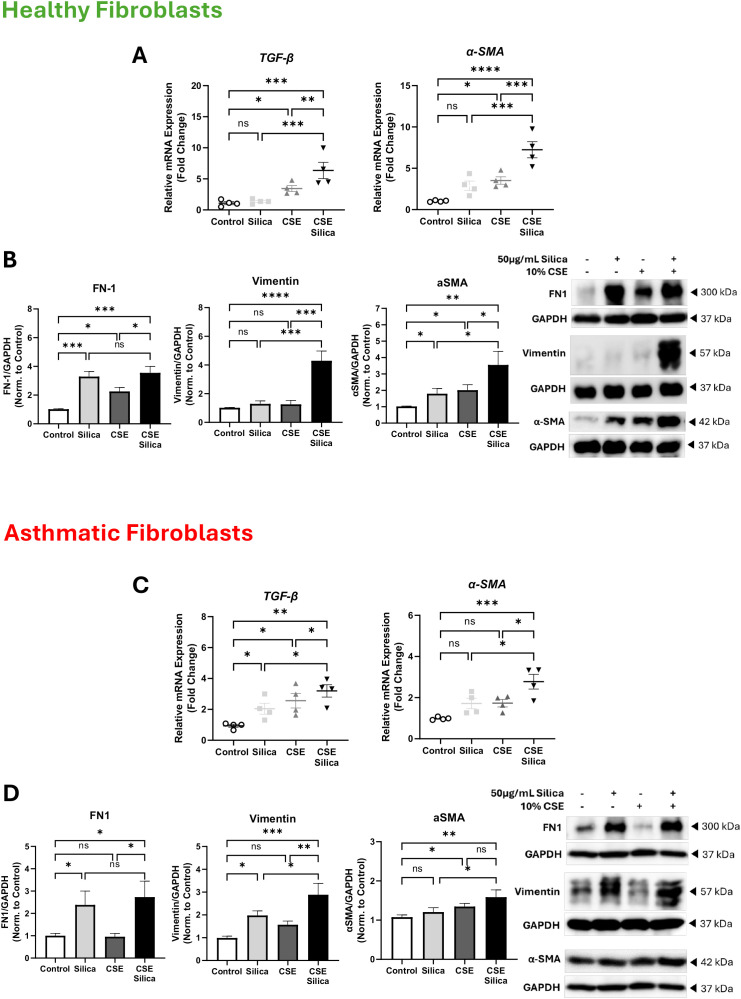
Cigarette smoke enhances the expression of fibrotic markers in silica-stimulated lung fibroblasts. **(A)** Healthy lung fibroblasts were stimulated with CSE and silica, then gene expression of TGF-β and α-SMA was compared relative to the unstimulated control. Data representative of 2 independent experiments from 2 unique donors in each group. **(B)** Representative western blot analysis and densitometric analysis of fibrotic mediators, including FN-1, Vimentin, and α-SMA, in healthy lung fibroblasts. GAPDH was used as a loading control. Data representative of three unique donors. Full blots are supplemented in **Fig.A in** S1 File. **(C)** Fibrotic markers were assessed following combined exposure to CSE and silica, and then the gene expression of TGF-β and α-SMA was compared to the unstimulated control in asthmatic lung fibroblasts. Data representative of 2 independent experiments from 2 unique donors in each group. **(D)** Representative western blot analysis and densitometric analysis of fibrotic mediators, including FN-1, Vimentin, and α-SMA in asthmatic lung fibroblasts. GAPDH was used as a loading control. Data representative of three unique donors. Full blots are supplemented in **Fig B in** S1 File. Results are presented as mean (± SEM) and relative to the control. The values were compared across the different groups using one-way ANOVA with a Bonferroni post hoc test for multiple comparisons. ns, non-significant. *p < 0.05, **p < 0.01, ***p < 0.001, ****p < 0.0001.

One of the main effects of silica and cigarette smoke exposure on lung tissue is the activation of fibroblasts, leading to the production of excessive amounts of extracellular matrix (ECM) components [29,30]. Here, we compared the effects of a combination of CSE and silica with those of unstimulated control, silica alone, and CSE alone in both healthy and asthmatic fibroblasts. Exposure of healthy fibroblasts to silica failed to induce the upregulation of *TGF-β1* and *α-SMA*. In contrast, a combined exposure to CSE and silica resulted in a significant upregulation of *TGF-β1* and *α-SMA* by 5.0-fold (from 1.4 to 6.4, p < 0.001) and 4.3-fold (from 2.9 to 7.2, p < 0.001), respectively, when compared to silica exposure alone. (Fig 2A). Data representative of 2 independent experiments from 2 unique donors in each group.

Furthermore, at the protein levels, FN-1 and α-SMA were increased upon silica stimulation for 24 h by 2.3-fold (from 1.0 to 3.3, p < 0.001) and 0.8-fold (from 1.0 to 1.8, p < 0.05), respectively, in comparison to the unstimulated control (Fig 2B). Upon exposure of silica-stimulated healthy fibroblasts to CSE, FN-1 protein showed a slight increase by 0.3-fold (from 3.3 to 3.6), while α-SMA protein was further increased by 1.8-fold (from 1.8 to 3.6, p < 0.05), compared to silica alone (Fig 2B). Vimentin was not induced by cigarette smoke or silica alone; however, the combination of these stimuli increased vimentin by 3-fold (from 1.3 to 4.3, p < 0.001) compared to silica alone. Data representative of three unique donors.

We next investigated whether a combination of silica and cigarette smoke would have a similar effect on lung fibroblasts isolated from asthmatic patients. Exposure to silica for 24 h in asthmatic fibroblasts resulted in the upregulation of the gene expression of *TGF-β1* by 1.1-fold (from 0.9 to 2.0, p < 0.05) relative to the unstimulated control, while there was no significant change in *α-SMA* mRNA level (Fig 2C). Similar to healthy fibroblasts, TGF-β1 and α-SMA gene expression was significantly enhanced upon combined exposure in asthmatic fibroblasts. The upregulated *TGF-β1* and *α-SMA* genes was significantly enhanced by 1.2-fold (from 2.0 to 3.2, p < 0.05) and 1.1-fold (from 1.7 to 2.8, p < 0.05), respectively, in asthmatic fibroblasts exposed to a combination of cigarette smoke and silica compared to silica alone (Fig 2C). Data representative of 2 independent experiments from 2 unique donors in each group.

The effect of the silica and cigarette smoke exposure was also confirmed at the protein level. Exposure to silica for 24 h in asthmatic fibroblasts resulted in the upregulation of the protein expression of FN1 by 1.4-fold (from 1.0 to 2.4, p < 0.05) and Vimentin by 1-fold (from 1.0 to 2.0, p < 0.05) relative to the unstimulated control, while there was no significant change in α-SMA protein level (Fig 2D). Interestingly, increases in FN1 didn’t change in combination with smoking and silica exposure, but the protein level of α-SMA was elevated by 0.4-fold (from 1.2 to 1.6, p < 0.05), and Vimentin was elevated by 0.9-fold (from 2.0 to 2.9, p < 0.05) compared to silica exposure alone (Fig 2D). Data representative of three unique donors.

### Exposure to both cigarette smoke and silica significantly induces the activation of matrix metalloproteinases and collagen deposition in asthmatic lung fibroblasts

**Fig 3:**
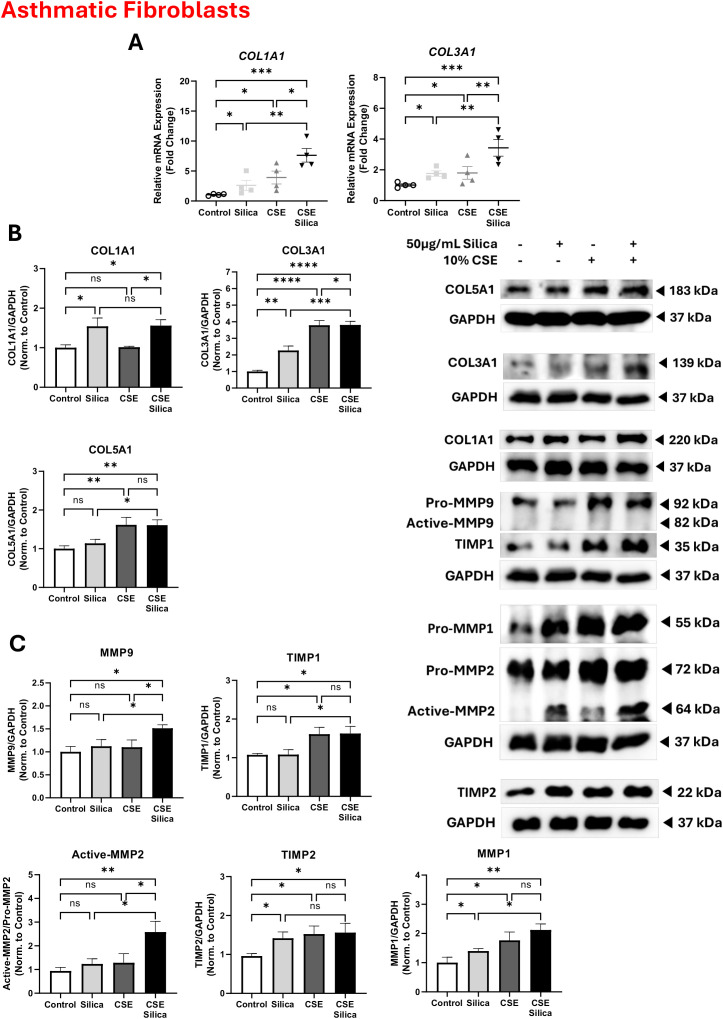
Exposure to both cigarette smoke and silica significantly induces the activation of matrix metalloproteinases and collagen deposition in asthmatic lung fibroblasts. **(A)** Comparison of *COL1A1* and *COL3A1* gene expression upon stimulation with CSE and silica in asthmatic lung fibroblasts. Data representative of 2 independent experiments from 2 unique donors in each group. Representative western blot analysis and densitometric analysis of markers of **(B)** collagen deposition (COL1A1, COL3A1, and COL5A1), **(C)** matrix metalloproteinases (MMPs) including MMP1, MMP2, and MMP9, and MMPs inhibitors including TIMP1 and TIMP2. GAPDH was used as a loading control. Data representative of three unique donors. Full blots for collagen deposition are supplemented in **Fig C in** S1 File, and for matrix metalloproteinases and their inhibitors are supplemented in **Fig D in** S1 File. Results are presented as mean (± SEM) and relative to the control. The values were compared across the different groups using one-way ANOVA with a Bonferroni post hoc test for multiple comparisons. ns, non-significant. *p < 0.05, **p < 0.01, ***p < 0.001, ****p < 0.0001.

To investigate the effects of cigarette smoke, silica, and their combination on matrix metalloproteinases (MMPs) and collagen deposition, protein levels were measured for MMP9, MMP2, and MMP1, as well as the MMP inhibitors TIMP1 and TIMP2, and the collagen markers COL1A1, COL3A1, and COL5A1. Exposure to silica for 24 h in asthmatic fibroblasts resulted in the upregulation of the gene expression of *COL1A1* by 1.6-fold (from 1.0 to 2.6, p < 0.05) and *COL3A1* by 0.8-fold (from 1.0 to 1.8, p < 0.05) relative to the unstimulated control (Fig 3A). The upregulation of *COL1A1* and *COL3A1* genes was significantly enhanced by 5-fold (from 2.6 to 7.6, p < 0.05) and 1.6-fold (from 1.8 to 3.4, p < 0.05), respectively, in asthmatic fibroblasts exposed to a combination of cigarette smoke and silica compared to silica alone (Fig 3A). Data representative of 2 independent experiments from 2 unique donors in each group.

The effect of the silica and cigarette smoke exposure was also confirmed at the protein level. Exposure to silica significantly increased COL1A1 by 0.5-fold (from 1.0 to 1.5, p < 0.05) and COL3A1 by 1.3-fold (from 1.0 to 2.3, p < 0.05), with no change in COL5A1 compared to unstimulated asthmatic fibroblasts. The combination of cigarette smoke and silica further increased COL3A1 by 1.5-fold (from 2.3 to 3.8, p < 0.05) and COL5A1 by 0.5-fold (from 1.1 to 1.6, p < 0.05) compared to silica alone (Fig 3B). Data representative of three unique donors.

Exposure of asthmatic fibroblasts to silica led to MMP2 activation compared to the control, which was further significantly increased by an additional 1.4-fold (from 1.2 to 2.6, p < 0.05) when fibroblasts were exposed to a combination of silica and cigarette smoke (Fig 3C). Additionally, silica stimulation did not upregulate MMP9 and MMP1 protein levels. While the combination of silica and CSE upregulated MMP9 and MMP1 protein levels by 0.4-fold (from 1.1 to 1.5, p < 0.05) and 0.7-fold (from 1.4 to 2.1, p < 0.05), respectively, compared to silica alone (Fig 3C). These findings suggest that the combination of silica and cigarette smoke synergistically enhances MMP activation in asthmatic fibroblasts. Upon stimulating asthmatic fibroblasts with silica, TIMP-2 protein levels increased by 0.4-fold (from 1.0 to 1.4, p < 0.05), while TIMP-1 levels remained unchanged. However, the combination of silica and cigarette smoke extract upregulated TIMP-1 by 0.5-fold (from 1.1 to 1.6, p < 0.05), without further increasing TIMP-2 levels compared to silica alone in asthmatic fibroblasts (Fig 3C). Data representative of three unique donors.

## Discussion

Cigarette smoke enhanced the fibrotic effects of silica in healthy and asthmatic lung fibroblasts. Healthy fibroblasts showed low baseline expression of fibrotic proteins but exhibited significant increases in ECM markers, including TGF-β1, α-SMA, FN-1, and Vimentin when exposed to CSE and silica. Asthmatic fibroblasts, with higher baseline levels of these markers, showed even greater upregulation upon exposure. The combination of silica and cigarette smoke also promoted collagen deposition and activated MMPs and their inhibitors in asthmatic fibroblasts. While previous studies have examined the effects of cigarette smoking and silica on healthy lung tissue [31], this is the first study to examine their combined effect on asthmatic fibroblasts, highlighting a pronounced profibrotic response in chronic inflammatory diseases.

Silica and cigarette smoke exert distinct effects on lung fibrotic markers. For instance, silica exposure resulted in an increase in the expression of fibrotic markers, such as TGF-β, αSMA, vimentin, and FN1, [29,30] mainly via the activation of fibroblasts [32]. Conversely, exposure to CSE resulted in obstructive changes in small airways, alveolar enlargement, and a progressive reduction in lung tissue resistance and elastance, primarily by upregulating TGF-β expression [33], increasing COL1A1 expression [34–36], and disrupting FN1 assembly [37]. Following our previous findings, we demonstrated that silica exposure induces fibrosis by increasing the expression of ECM proteins, including TGF-β, α-SMA, vimentin, and fibronectin, in asthmatic fibroblasts (Fig 2C). Interestingly, exposing fibroblasts isolated from both healthy and asthmatic individuals to a combination of silica and cigarette smoke results in an additional increase in fibrotic markers (Fig 2). Another key mediator for fibrosis is the activation of MMPs. Silica exposure has been shown to induce MMP2 [38] and MMP9 [38,39]. Further, CSE exerts a direct effect on the proteinase/anti-proteinase balance, which resulted in a significant increase in MMP2 [36,40], MMP1 [36,41], and MMP9 [42] expression and a dysregulated TIMP expression [36,40]. Our data are consistent with previous findings, indicating that silica stimulation significantly increases the expression of MMP1 and MMP9, as well as the activation of MMP2, in asthmatic fibroblasts (Fig 3C). Notably, exposure to cigarette smoke induces additional effects on MMPs following silica exposure (Fig 3C).

The combined effects of silica exposure and cigarette smoking have been linked to increased mortality in individuals with lung cancer, specific infectious and parasitic diseases, respiratory tuberculosis, respiratory system diseases, and pneumoconiosis [20]. A study with 16,901 cases and 20,965 control subjects found that silica exposure is linked to a cumulative increase in lung cancer risk, even at low levels [43]. Additionally, the combination of cigarette smoke and silica exposure significantly raises the likelihood of developing lung cancer [43].

Although the combination of silica and smoking in otherwise healthy individuals is a risk factor for lung fibrotic, inflammatory, and neoplastic disorders, this complication is enhanced in patients with underlying lung conditions like COPD and asthma [44]. An examination of 2,209 white South African gold miners aged 45–54 revealed a relative risk of 2.5 for COPD linked to silica dust exposure [44]. The results indicated that silica dust and cigarette smoking had a synergistic effect, with all miners who died from COPD being smokers. Approximately 59% of COPD-related deaths were attributed to the combined effects of silica dust and smoking, emphasizing the increased risk for miners exposed to both hazards [44]. In our study, similarly, the increase in profibrotic markers in response to exposure to a combination of silica and cigarette smoke was more enhanced in asthmatic than in healthy fibroblasts. Notably, this is the first study to examine the synergistic fibrotic effects of cigarette smoke and silica in lung fibroblasts isolated from asthmatic individuals, addressing a key knowledge gap in occupational and environmental health. The findings could inform the development of targeted interventions and revisions to occupational safety standards, particularly in regions with high environmental dust exposure or prevalent smoking habits. Cessation of smoking and control of silica exposure are essential for reducing lung inflammation and fibrosis. In addition, novel agents such as DNase I [45] and calprotectin inhibitors [46], and existing treatments such as vitamin D [47,48] and statins [49] have shown promise in reducing the associated risks and warrant further investigation.

## Conclusion

In summary, our findings contribute to a better understanding of the complex pathogenesis of pulmonary fibrosis. They emphasize the importance of considering environmental exposures, such as cigarette smoke and silica, in the development and progression of lung fibrosis, particularly in individuals with asthma. These insights emphasize the need for stricter occupational regulations and smoking cessation programs, especially in dust-exposed environments. Furthermore, this is the first study to evaluate the combined impact of silica and cigarette smoke on asthmatic fibroblasts, providing novel evidence that may influence both clinical and public health policies aimed at minimizing environmental risk factors in respiratory diseases. Further research is warranted to elucidate the underlying mechanisms and therapeutic implications of these interactions for the management of fibrotic lung diseases.

## Supporting information

S1 File Raw images. (PDF)

## Abbreviations

α-SMA: α-Smooth Muscle Actin
COL1A1: Collagen type I alpha 1 chain
CSE: Cigarette smoke extract
FN1: Fibronectin 1
MMP1: Matrix Metalloproteinase 1
MMP2: Matrix Metalloproteinase 2
MMP9: Matrix Metalloproteinase 9
TIMP-2: TIMP metallopeptidase inhibitor 2
TGF-β: Transforming Growth Factor-β

## References

[1] Awadh SM. Geochemistry and mineralogical composition of the airborne particles of sand dunes and dust storms settled in Iraq and their environmental impacts. Environ Earth Sci. 2011;66(8):2247–56. doi: 10.1007/s12665-011-1445-6

[2] Howari FM, Baghdady A, Goodell PC. Mineralogical and gemorphological characterization of sand dunes in the eastern part of United Arab Emirates using orbital remote sensing integrated with field investigations. Geomorphology. 2007;83(1–2):67–81. doi: 10.1016/j.geomorph.2006.06.015

[3] Occupational Safety and Health Administration. OSHA’s proposed crystalline silica rule: overview. 2013. https://www.osha.gov/silica/factsheets/OSHA_FS-3683_Silica_Overview.pdf

[4] ’t Mannetje A, Steenland K, Attfield M, Boffetta P, Checkoway H, DeKlerk N, et al. Exposure-response analysis and risk assessment for silica and silicosis mortality in a pooled analysis of six cohorts. Occup Environ Med. 2002;59(11):723–8. doi: 10.1136/oem.59.11.723 12409529 PMC1740236

[5] Lenander-Ramirez A, Bryngelsson I-L, Vihlborg P, Westberg H, Andersson L. Respirable dust and silica: respiratory diseases among swedish iron foundry workers. J Occup Environ Med. 2022;64(7):593–8. doi: 10.1097/JOM.0000000000002533 35275887 PMC9301988

[6] Picciotto S, Neophytou AM, Brown DM, Checkoway H, Eisen EA, Costello S. Occupational silica exposure and mortality from lung cancer and nonmalignant respiratory disease: G-estimation of structural nested accelerated failure time models. Environ Epidemiol. 2018;2(3):e029. doi: 10.1097/EE9.0000000000000029 33210072 PMC7660981

[7] Makrufardi F, Manullang A, Rusmawatiningtyas D, Chung KF, Lin S-C, Chuang H-C. Extreme weather and asthma: a systematic review and meta-analysis. Eur Respir Rev. 2023;32(168):230019. doi: 10.1183/16000617.0019-2023 37286218 PMC10245140

[8] D’Amato G, Akdis CA. Desert dust and respiratory diseases: further insights into the epithelial barrier hypothesis. Allergy. 2022;77(12):3490–2. doi: 10.1111/all.15392 35633073

[9] Meo SA, Al-Kheraiji MFA, Alfaraj ZF, Alwehaibi NA, Aldereihim AA. Respiratory and general health complaints in subjects exposed to sandstorm at Riyadh, Saudi Arabia. Pak J Med Sci. 2013;29(2):642–6. doi: 10.12669/pjms.292.3065 24353595 PMC3809255

[10] Kanatani KT, Ito I, Al-Delaimy WK, Adachi Y, Mathews WC, Ramsdell JW, et al. Desert dust exposure is associated with increased risk of asthma hospitalization in children. Am J Respir Crit Care Med. 2010;182(12):1475–81. doi: 10.1164/rccm.201002-0296OC 20656941 PMC3159090

[11] Thalib L, Al-Taiar A. Dust storms and the risk of asthma admissions to hospitals in Kuwait. Sci Total Environ. 2012;433:347–51. doi: 10.1016/j.scitotenv.2012.06.082 22819885

[12] Mahboub BH, Al-Hammadi S, Rafique M, Sulaiman N, Pawankar R, Al Redha AI, et al. Population prevalence of asthma and its determinants based on European Community Respiratory Health Survey in the United Arab Emirates. BMC Pulmonary Medicine. 2012;12(1):null. doi: 10.1186/1471-2466-12-4 PMC3306751 22340199

[13] Thomson NC, Polosa R, Sin DD. Cigarette smoking and asthma. J Allergy Clin Immunol Pract. 2022;10(11):2783–97. doi: 10.1016/j.jaip.2022.04.034 35533997

[14] Shaw DE, Sousa AR, Fowler SJ, Fleming LJ, Roberts G, Corfield J, et al. Clinical and inflammatory characteristics of the European U-BIOPRED adult severe asthma cohort. Eur Respir J. 2015;46(5):1308–21. doi: 10.1183/13993003.00779-2015 26357963

[15] Butz AM, Tsoukleris M, Elizabeth Bollinger M, Jassal M, Bellin MH, Kub J, et al. Association between second hand smoke (SHS) exposure and caregiver stress in children with poorly controlled asthma. J Asthma. 2019;56(9):915–26. doi: 10.1080/02770903.2018.1509989 30307351 PMC6551304

[16] Neophytou AM, Oh SS, White MJ, Mak ACY, Hu D, Huntsman S, et al. Secondhand smoke exposure and asthma outcomes among African-American and Latino children with asthma. Thorax. 2018;73(11):1041–8. doi: 10.1136/thoraxjnl-2017-211383 29899038 PMC6225993

[17] Gopal SH, Mukherjee S, Das SK. Direct and Second Hand Cigarette Smoke Exposure and Development of Childhood Asthma. J Environ Health Sci. 2016;2(6). doi: 10.15436/2378-6841.16.1122 29399637 PMC5791751

[18] Eisner MD, Klein J, Hammond SK, Koren G, Lactao G, Iribarren C. Directly measured second hand smoke exposure and asthma health outcomes. Thorax. 2005;60(10):814–21. doi: 10.1136/thx.2004.037283 16192366 PMC1747192

[19] Boulet L-P, Lemière C, Archambault F, Carrier G, Descary MC, Deschesnes F. Smoking and asthma: clinical and radiologic features, lung function, and airway inflammation. Chest. 2006;129(3):661–8. doi: 10.1378/chest.129.3.661 16537865

[20] Wang D, Yang M, Liu Y, Ma J, Shi T, Chen W. Association of silica dust exposure and cigarette smoking with mortality among mine and pottery workers in China. JAMA Netw Open. 2020;3(4):e202787. doi: 10.1001/jamanetworkopen.2020.2787 32286660 PMC7156992

[21] Lai H, Liu Y, Zhou M, Shi T, Zhou Y, Weng S, et al. Combined effect of silica dust exposure and cigarette smoking on total and cause-specific mortality in iron miners: a cohort study. Environ Health. 2018;17(1):46. doi: 10.1186/s12940-018-0391-0 29743082 PMC5943994

[22] Tse LA, Yu ITS, Qiu H, Leung CC. Joint effects of smoking and silicosis on diseases to the lungs. PLoS One. 2014;9(8):e104494. doi: 10.1371/journal.pone.0104494 25105409 PMC4126694

[23] Hessel PA, Gamble JF, Nicolich M. Relationship between silicosis and smoking. Scand J Work Environ Health. 2003;29(5):329–36. doi: 10.5271/sjweh.739 14584513

[24] Sager TM, Umbright CM, Mustafa GM, Yanamala N, Leonard HD, McKinney WG, et al. Tobacco smoke exposure exacerbated crystalline silica-induced lung toxicity in rats. Toxicol Sci. 2020;178(2):375–90. doi: 10.1093/toxsci/kfaa146 32976597 PMC7825013

[25] Handra C-M, Gurzu I-L, Chirila M, Ghita I. Silicosis: new challenges from an old inflammatory and fibrotic disease. Front Biosci (Landmark Ed). 2023;28(5):96. doi: 10.31083/j.fbl2805096 37258484

[26] Bellou V, Belbasis L, Evangelou E. Tobacco smoking and risk for pulmonary fibrosis: a prospective cohort study from the UK biobank. Chest. 2021;160(3):983–93. doi: 10.1016/j.chest.2021.04.035 33905677

[27] Wenzel SE. Asthma phenotypes: the evolution from clinical to molecular approaches. Nat Med. 2012;18(5):716–25. doi: 10.1038/nm.2678 22561835

[28] Mdkhana B, Saheb Sharif-Askari N, Ramakrishnan RK, Al-Sheakly BK, Hafezi S, Saheb Sharif-Askari F, et al. Nucleic acid sensor STING drives remodeling and its inhibition enhances steroid responsiveness in chronic obstructive pulmonary disease. PLoS One. 2023;18(7):e0284061. doi: 10.1371/journal.pone.0284061 37406004 PMC10321631

[29] Yuan L, Sun Y, Zhou N, Wu W, Zheng W, Wang Y. Dihydroquercetin attenuates silica-induced pulmonary fibrosis by inhibiting ferroptosis signaling pathway. Front Pharmacol. 2022;13:845600. doi: 10.3389/fphar.2022.845600 35645837 PMC9133504

[30] Ali SA, Saifi MA, Godugu C, Talla V. Silibinin alleviates silica-induced pulmonary fibrosis: potential role in modulating inflammation and epithelial-mesenchymal transition. Phytother Res. 2021;35(9):5290–304. doi: 10.1002/ptr.7210 34250649

[31] Chen H, Tao X, Cao H, Li B, Sun Q, Wang W, et al. Nicotine exposure exacerbates silica-induced pulmonary fibrosis via STAT3-BDNF-TrkB-mediated epithelial-mesenchymal transition in alveolar type II cells. Food Chem Toxicol. 2023;175:113694. doi: 10.1016/j.fct.2023.113694 36868510

[32] Li S, Li C, Zhang Y, He X, Chen X, Zeng X, et al. Targeting mechanics-induced fibroblast activation through CD44-RhoA-YAP pathway ameliorates crystalline silica-induced silicosis. Theranostics. 2019;9(17):4993–5008. doi: 10.7150/thno.35665 31410197 PMC6691376

[33] Takizawa H, Tanaka M, Takami K, Ohtoshi T, Ito K, Satoh M, et al. Increased expression of transforming growth factor-beta1 in small airway epithelium from tobacco smokers and patients with chronic obstructive pulmonary disease (COPD). Am J Respir Crit Care Med. 2001;163(6):1476–83. doi: 10.1164/ajrccm.163.6.9908135 11371421

[34] Junqueira JJM, Lourenço JD, da Silva KR, Cervilha DA de B, da Silveira LKR, Correia AT, et al. Decreased bone type i collagen in the early stages of chronic obstructive pulmonary disease (COPD). COPD. 2020;17(5):575–86. doi: 10.1080/15412555.2020.1808605 32814449

[35] Overbeek SA, Braber S, Koelink PJ, Henricks PAJ, Mortaz E, LoTam Loi AT, et al. Cigarette smoke-induced collagen destruction; key to chronic neutrophilic airway inflammation?. PLoS One. 2013;8(1):e55612. doi: 10.1371/journal.pone.0055612 23383243 PMC3561332

[36] Zhang W, Song F, Windsor LJ. Cigarette smoke condensate affects the collagen-degrading ability of human gingival fibroblasts. J Periodontal Res. 2009;44(6):704–13. doi: 10.1111/j.1600-0765.2008.01179.x 19453854

[37] Xue J, Liao Q, Luo M, Hua C, Zhao J, Yu G, et al. Cigarette smoke-induced oxidative stress activates NRF2 to mediate fibronectin disorganization in vascular formation. Open Biol. 2022;12(4):210310. doi: 10.1098/rsob.210310 35472288 PMC9042581

[38] Scabilloni JF, Wang L, Antonini JM, Roberts JR, Castranova V, Mercer RR. Matrix metalloproteinase induction in fibrosis and fibrotic nodule formation due to silica inhalation. Am J Physiol Lung Cell Mol Physiol. 2005;288(4):L709-17. doi: 10.1152/ajplung.00034.2004 15608151

[39] Jiang P-R, Cao Z, Qiu Z-L, Pan J-W, Zhang N, Wu Y-F. Plasma levels of TNF-α and MMP-9 in patients with silicosis. Eur Rev Med Pharmacol Sci. 2015;19(9):1716–20. 26004615

[40] Park J-H, Shin J-M, Yang H-W, Kim TH, Lee SH, Lee H-M, et al. Cigarette smoke extract stimulates MMP-2 production in nasal fibroblasts via ROS/PI3K, Akt, and NF-κB signaling pathways. Antioxidants (Basel). 2020;9(8):739. doi: 10.3390/antiox9080739 32806646 PMC7465436

[41] Adam G, Shiomi T, Monica G, Jarrod S, Vincent A, Becky M, et al. Suppression of cigarette smoke induced MMP1 expression by selective serotonin re-uptake inhibitors. FASEB J. 2021;35(7):e21519. doi: 10.1096/fj.202001966RR 34137477 PMC9292461

[42] Zhou L, Le Y, Tian J, Yang X, Jin R, Gai X, et al. Cigarette smoke-induced RANKL expression enhances MMP-9 production by alveolar macrophages. Int J Chron Obstruct Pulmon Dis. 2018;14:81–91. doi: 10.2147/COPD.S190023 30587964 PMC6304243

[43] Ge C, Peters S, Olsson A, Portengen L, Schüz J, Almansa J, et al. Respirable crystalline silica exposure, smoking, and lung cancer subtype risks. a pooled analysis of case-control studies. Am J Respir Crit Care Med. 2020;202(3):412–21. doi: 10.1164/rccm.201910-1926OC 32330394 PMC7465090

[44] Hnizdo E. Combined effect of silica dust and tobacco smoking on mortality from chronic obstructive lung disease in gold miners. Br J Ind Med. 1990;47(10):656–64. doi: 10.1136/oem.47.10.656 2171628 PMC1012022

[45] Benmerzoug S, Rose S, Bounab B, Gosset D, Duneau L, Chenuet P, et al. STING-dependent sensing of self-DNA drives silica-induced lung inflammation. Nat Commun. 2018;9(1):5226. doi: 10.1038/s41467-018-07425-1 30523277 PMC6283886

[46] Saheb Sharif-Askari N, Mdkhana B, Hafezi S, Elamin OF, Eladham MW, Al-Sheakly BKS, et al. Calprotectin inhibition attenuates silica-induced lung fibrosis. Inflammopharmacology. 2025;33(5):2869–81. doi: 10.1007/s10787-025-01771-5 40381145

[47] Yang Y, Wei S, Li Q, Chu K, Zhou Y, Xue L, et al. Vitamin D protects silica particles induced lung injury by promoting macrophage polarization in a KLF4-STAT6 manner. J Nutr Biochem. 2022;110:109148. doi: 10.1016/j.jnutbio.2022.109148 36049670

[48] Koul A, Angmo S, Bharati S. Preventive role of vitamin D in silica-induced skin fibrosis: a study in relation to oxidative stress and pro-inflammatory cytokines. Int J Vitam Nutr Res. 2016;86(3–4):88–96. doi: 10.1024/0300-9831/a000434 29219782

[49] Bo C, Liu F, Zhang Z, Du Z, Xiu H, Zhang Z, et al. Simvastatin attenuates silica-induced pulmonary inflammation and fibrosis in rats via the AMPK-NOX pathway. BMC Pulm Med. 2024;24(1):224. doi: 10.1186/s12890-024-03014-9 38720270 PMC11080310

