# Supplementary material for: A combination of silica and cigarette smoke extract exacerbates lung fibrosis: Unveiling a harmful synergy
Source: PLoS One. 2025 Aug 22;20(8):e0330762. doi: 10.1371/journal.pone.0330762 (PMC12373220; doi:10.1371/journal.pone.0330762)

Figure A

Healthy Fibroblasts

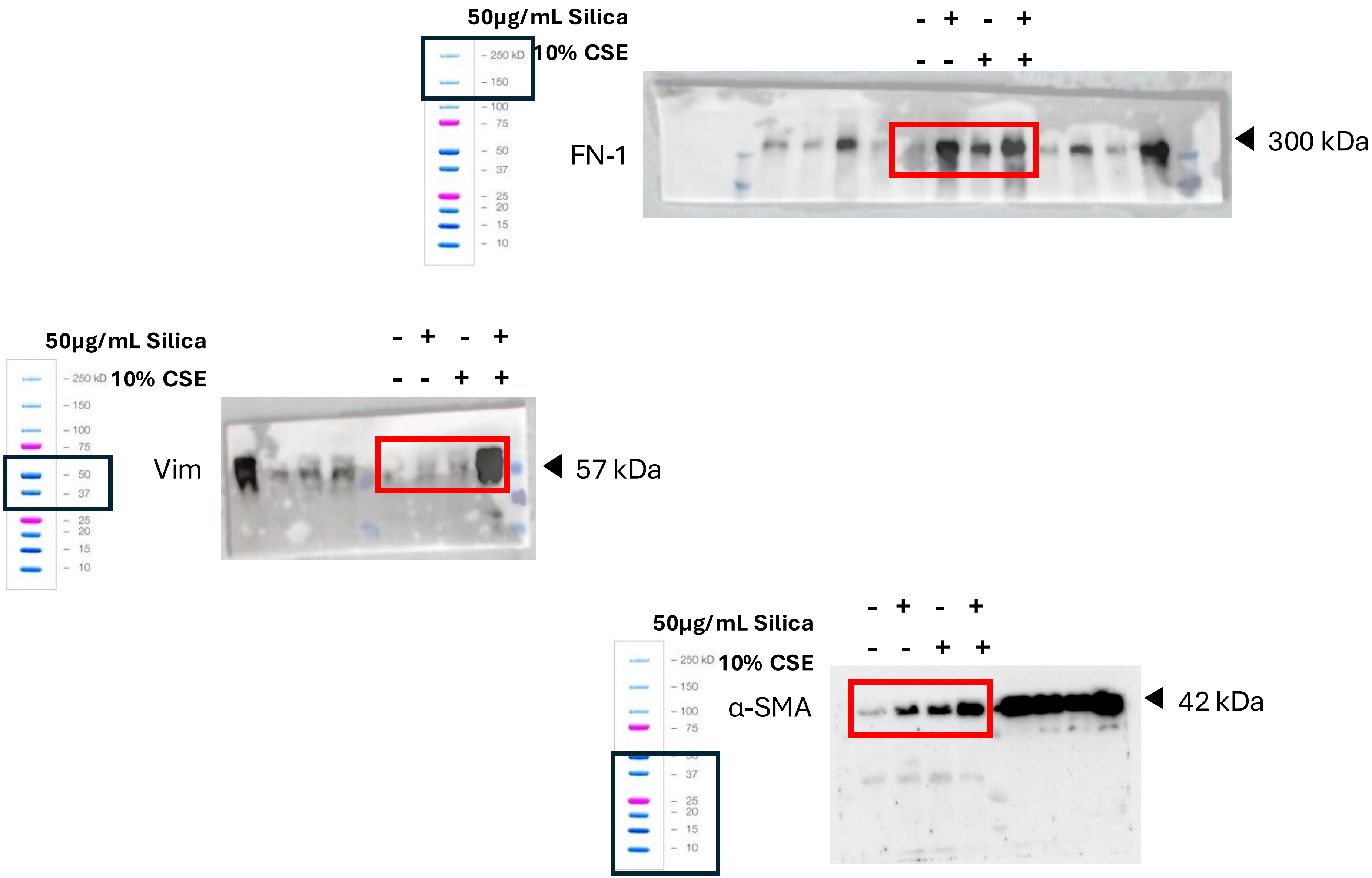

Figure B

Asthmatic Fibroblasts

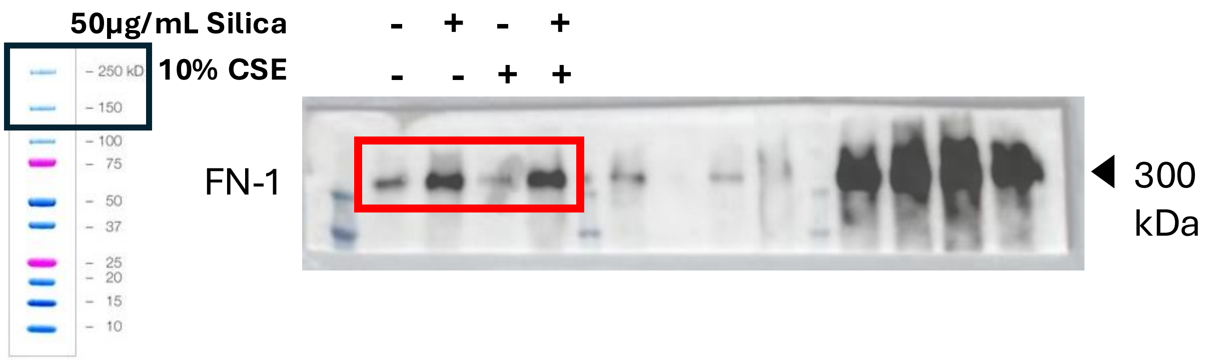

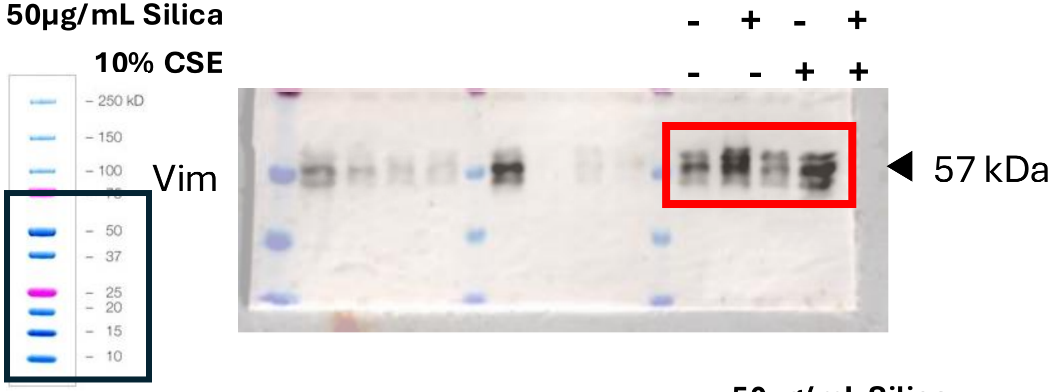

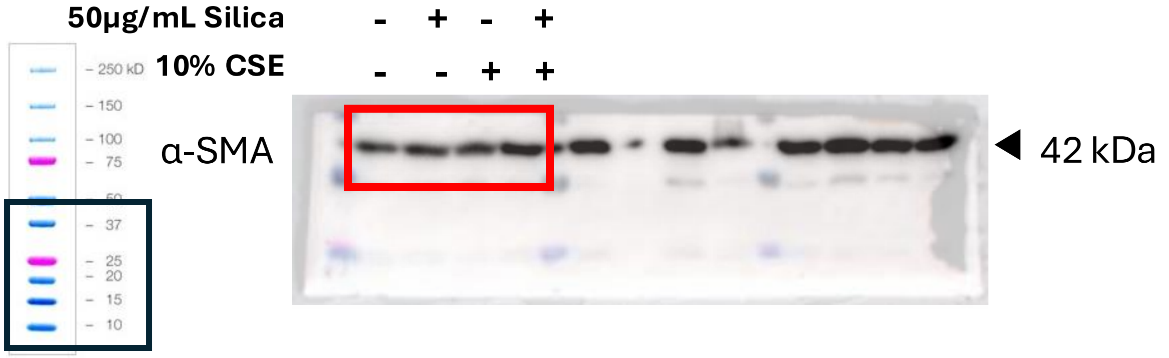

Figure C

Asthmatic Fibroblasts

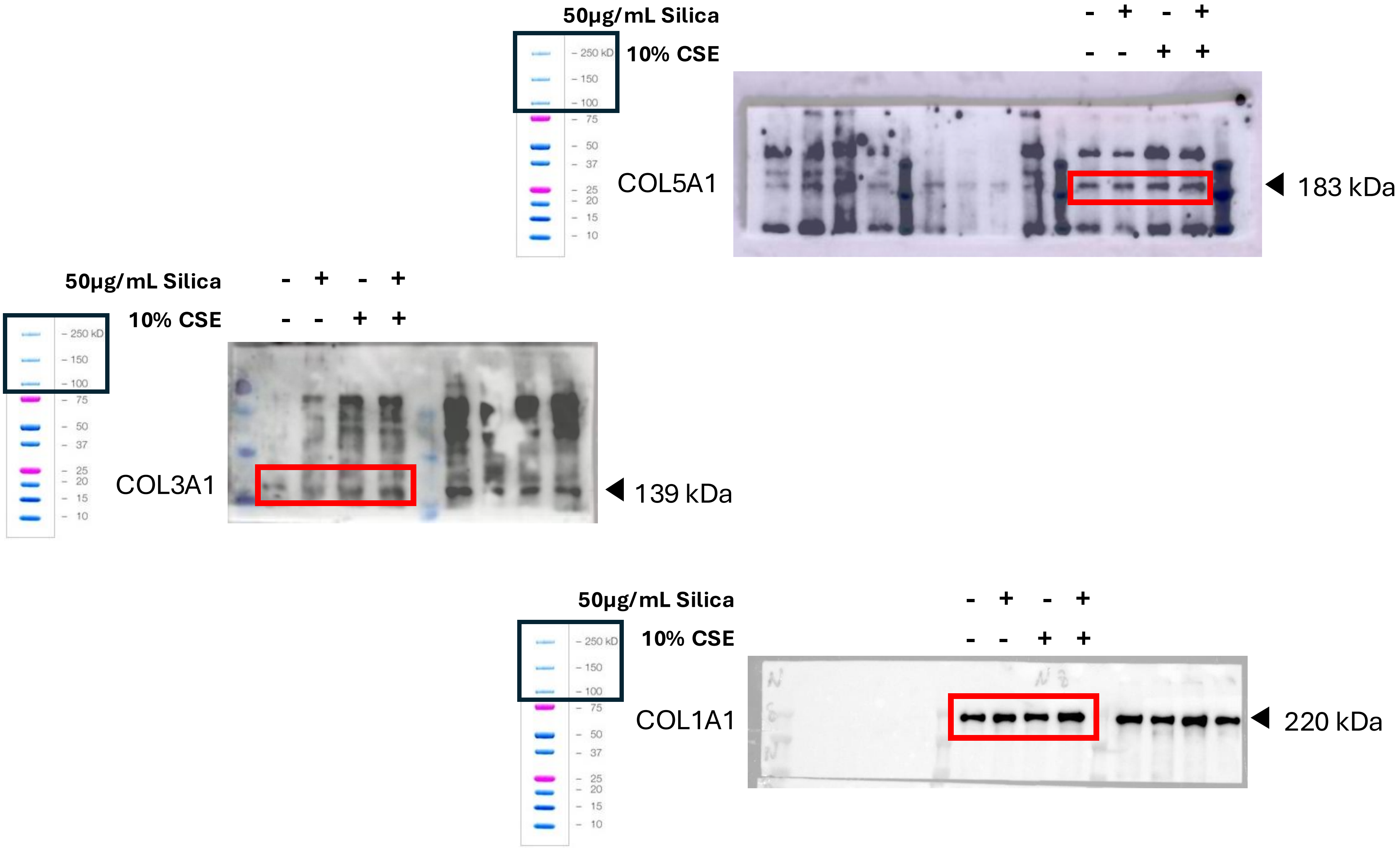

Figure D

Asthmatic Fibroblasts

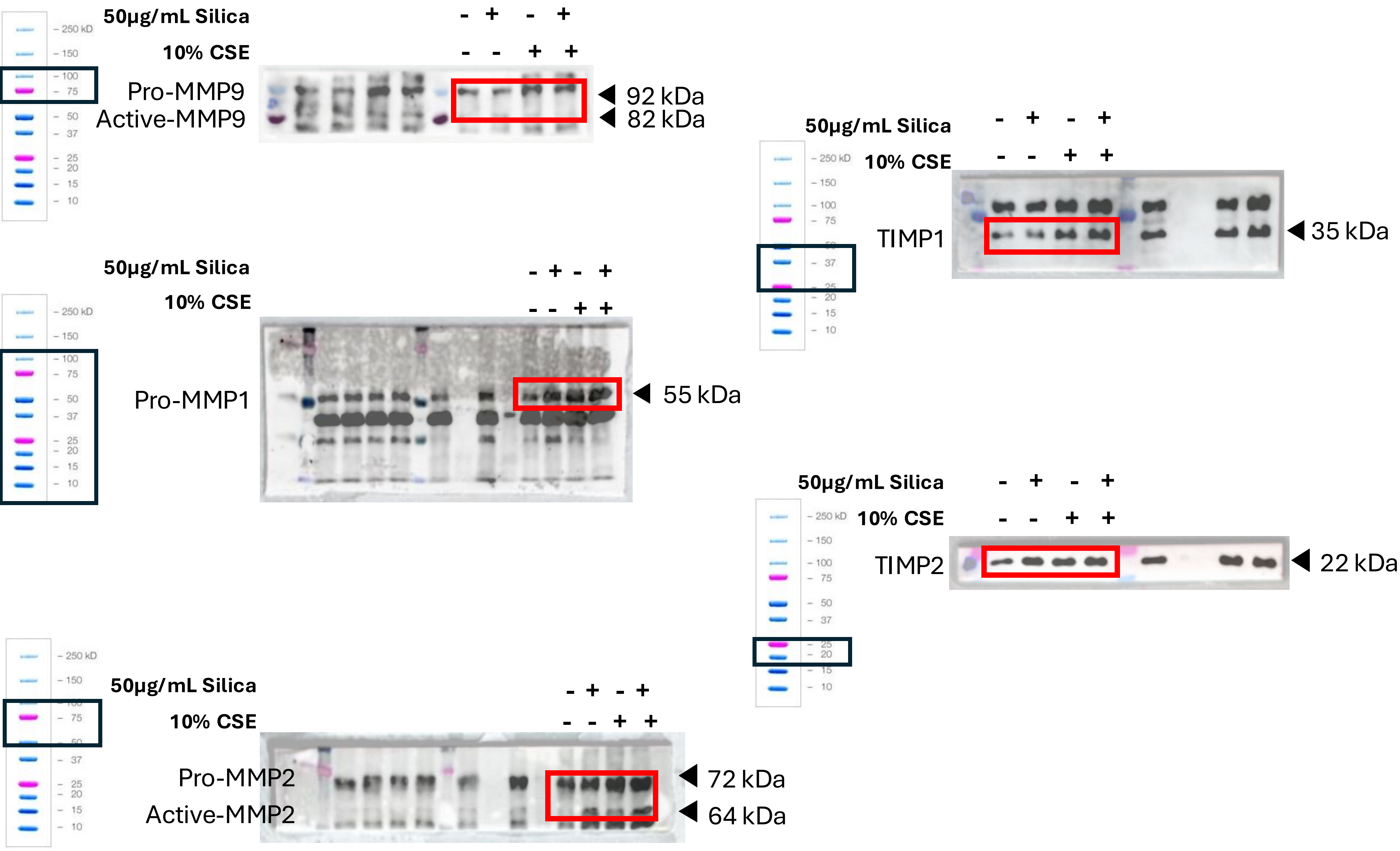

Supplement: S1 File — (PDF) [file pone.0330762.s001.pdf]
